# Supplementary material for: Long-term ambient hydrocarbon exposure and incidence of urinary bladder cancer
Source: Sci Rep. 2022 Dec 2;12:20799. doi: 10.1038/s41598-022-25425-6 (PMC9718740; doi:10.1038/s41598-022-25425-6)
Supplement: Supplementary file 1 — Supplementary Information. [file 41598_2022_25425_MOESM1_ESM.docx]

Supplementary Information

**Long-term ambient hydrocarbon exposure and incidence of urinary bladder cancer**

Han-Wei Zhang, PhD, Zhi-Ren Tsai, PhD, Victor C. Kok, MD, PhD, FACP, Hsiao-Ching Peng, MSc, Yau-Hung Chen, PhD, Jeffrey J.P. Tsai, PhD, and Chung Y. Hsu, PhD

Corresponding Author: Victor C. Kok, victorkok@asia.edu.tw

Supplementary Figures: 3.

Supplementary Tables: 4.

**Legends of Figures and Tables**

**Supplementary Figure S1.** Population distribution of the daily average concentrations of air pollutants (SO_2_, CO_2_, CO, O_3_, PM_10_, and PM_2.5_) over a 10-year exposure period.

**Supplementary Figure S2.** Population distribution of the daily average concentrations of air pollutants (NO_X_, NO, NO_2_, THC, NMHC, and CH_4_) over a 10-year exposure period.

**Supplementary Figure S3.** Both ambient pollutants show a long-term monotonic downward trend over time according to the Mann-Kendall test. Sen’s method used to estimate the slope of the trend over the study period revealed a downward trend of -0.03 units per year for THC (95% CI, -0.04 to -0.02; *** p < 0.001) and -0.01 units/year for NMHC (95% CI, -0.01 to -0.01; *** p < 0.001).

**Supplementary Table S1.** Pearson’s correlation analysis of air pollutants in the present study population at risk over a 10-year exposure period.

**Supplementary Table S2**. Mean and distribution of air pollutants linked to the present study population according to the postal code over a 10-year exposure period.

**Supplementary Table S3**. Sensitivity analysis showing adjusted hazard ratios of developing urinary bladder cancer stratified by sex during long-term THC or NMHC exposure at a standard deviation (SD) increment controlled for PM2.5 and other air pollutants

**Supplementary Table S4.** Sensitivity analysis showing adjusted hazard ratios of the incidence of urinary bladder cancer stratified by diabetes mellitus status during long-term THC or NMHC exposure at a standard deviation (SD) increment controlled for PM2.5 and other air pollutants.

**Supplementary Figure S1.** Population distribution of the daily average concentrations of air pollutants (SO_2_, CO_2_, CO, O_3_, PM_10_, and PM_2.5_) over a 10-year exposure period.


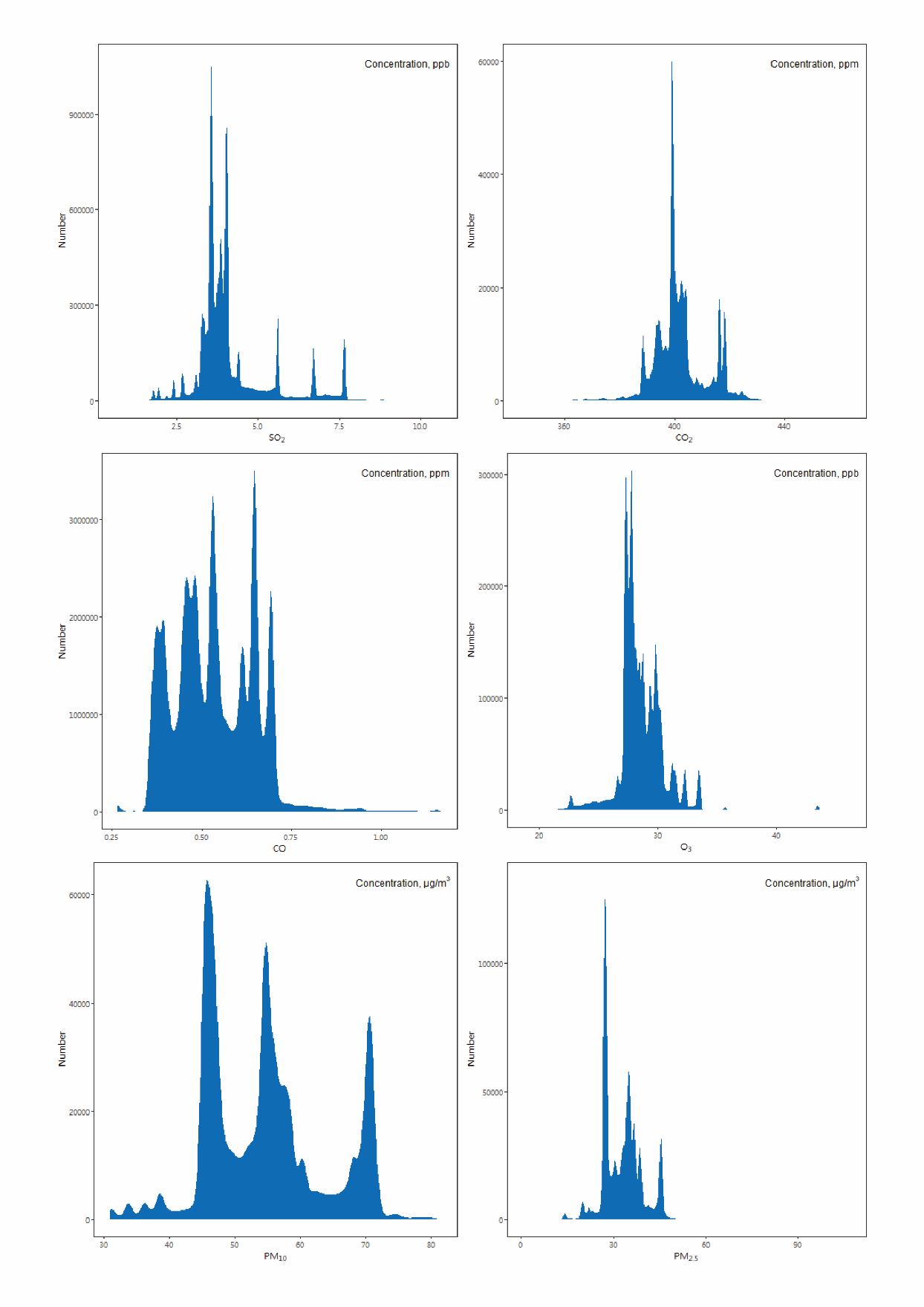


**Supplementary Figure S2.** Population distribution of the daily average concentrations of air pollutants (NO_X_, NO, NO_2_, THC, NMHC, and CH_4_) over a 10-year exposure period.

**
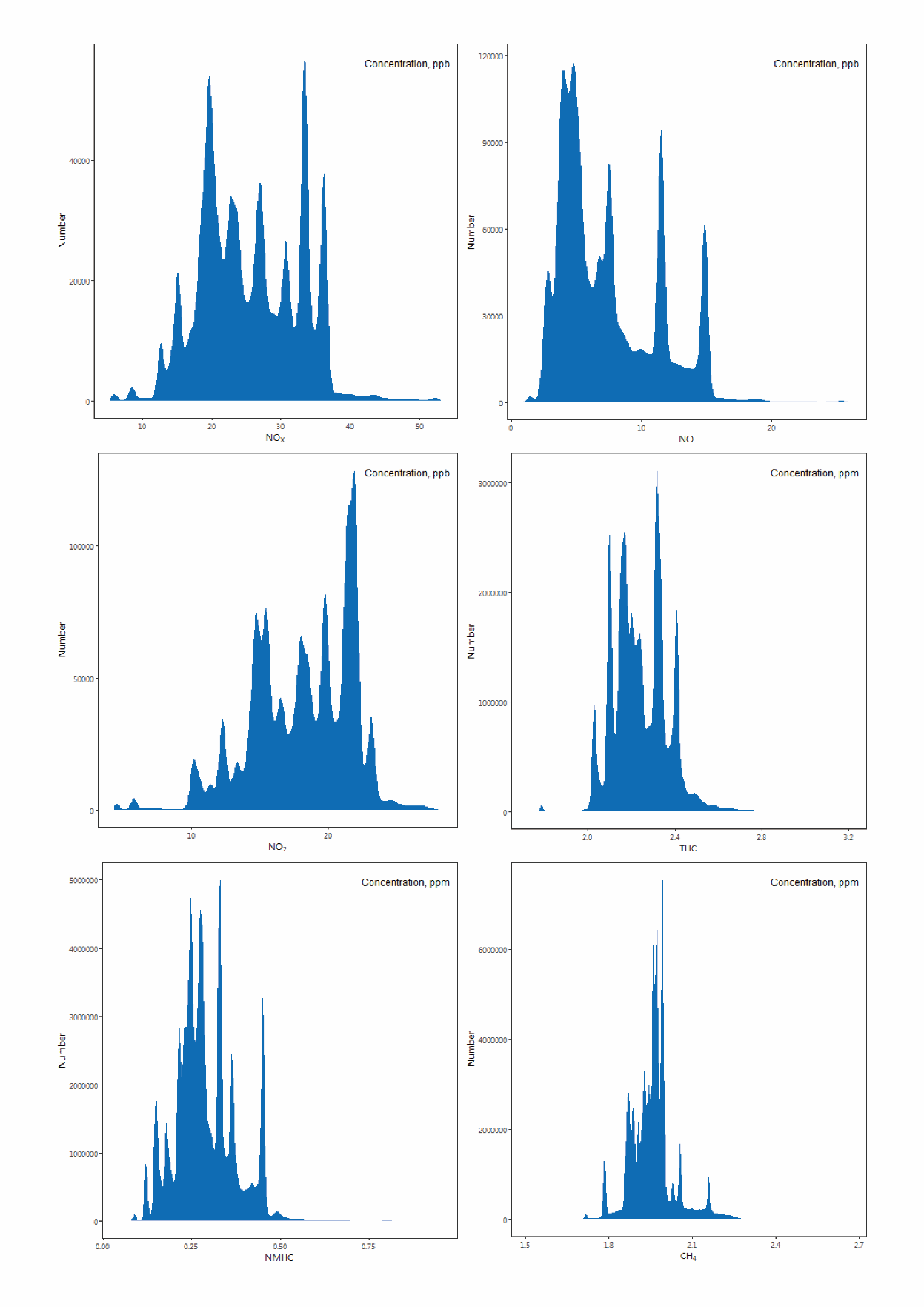
**

**Supplementary Figure S3.** Both ambient pollutants show a long-term monotonic downward trend over time according to the Mann-Kendall test.

**
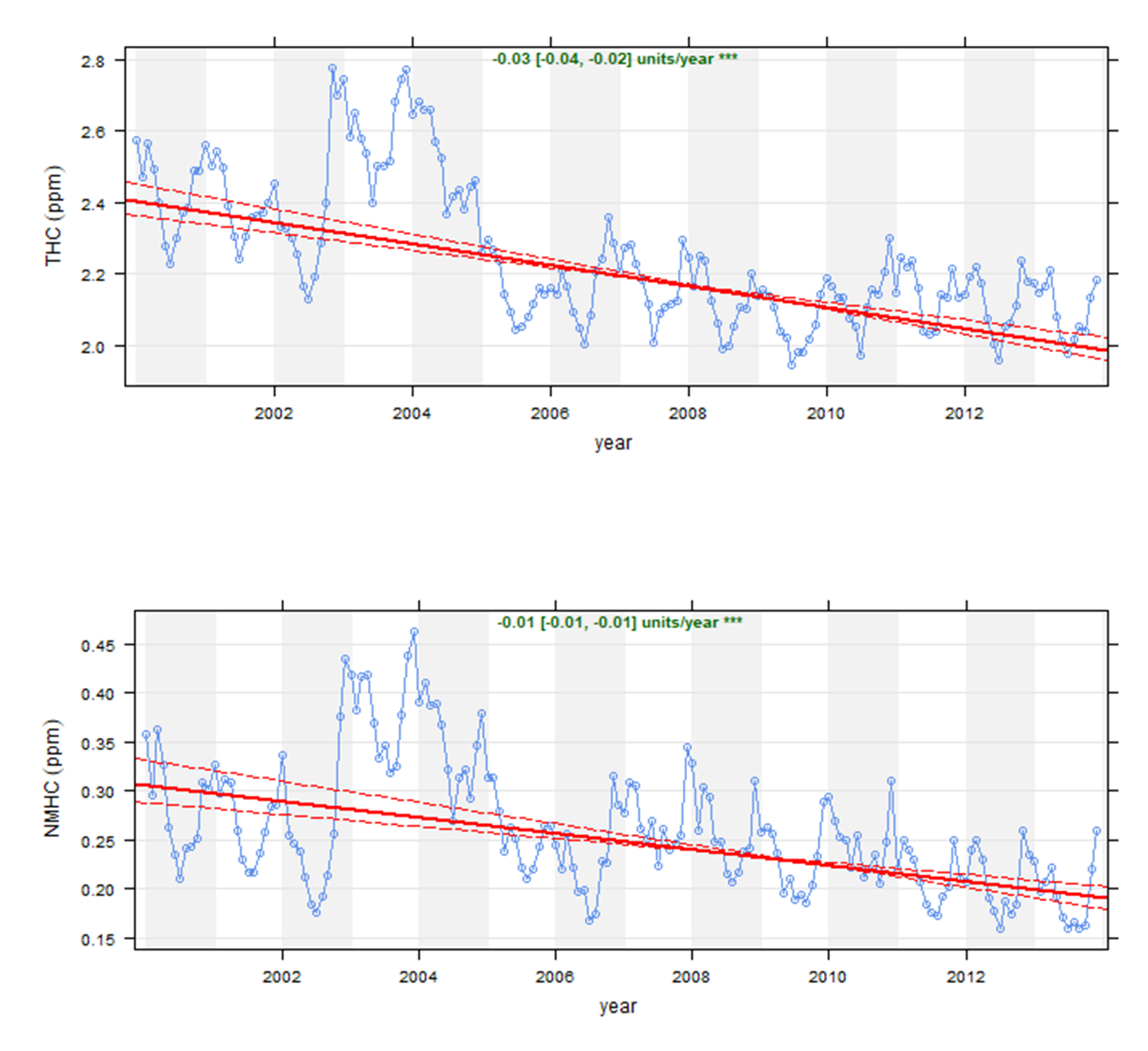
**

Sen’s method used to estimate the slope of the trend over the study period revealed a downward trend of -0.03 units per year for THC (95% CI, -0.04 to -0.02; *** p < 0.001) and -0.01 units/year for NMHC (95% CI, -0.01 to -0.01; *** p < 0.001).

**Supplementary Table S1.** Pearson’s correlation analysis of air pollutants in the present study population at risk over a 10-year exposure period

|  | SO_2_ | CO_2_ | CO | O_3_ | PM_10_ | PM_2.5_ | NO_X_ | NO | NO_2_ | THC | NMHC | CH_4_ |
| --- | --- | --- | --- | --- | --- | --- | --- | --- | --- | --- | --- | --- |
| SO_2_ | 1 | **-0.025^†^** | **0.196^†^** | **0.011^†^** | 0.612**^†^** | 0.602**^†^** | **0.266^†^** | **0.074^†^** | 0.441**^†^** | **0.089^†^** | **0.159^†^** | **-0.033^†^** |
| CO_2_ |  | 1 | **-0.286^†^** | **0.042^†^** | 0.380**^†^** | **0.262^†^** | **-0.224^†^** | **-0.299^†^** | **-0.130^†^** | -0.489**^†^** | -0.413**^†^** | -0.370**^†^** |
| CO |  |  | 1 | -0.638**^†^** | -0.306**^†^** | **-0.223^†^** | 0.960**^†^** | 0.930**^†^** | 0.890**^†^** | 0.687**^†^** | 0.865**^†^** | **0.183^†^** |
| O_3_ |  |  |  | 1 | 0.341**^†^** | **0.298^†^** | -0.587**^†^** | -0.524**^†^** | -0.592**^†^** | -0.436**^†^** | -0.483**^†^** | **-0.186^†^** |
| PM_10_ |  |  |  |  | 1 | 0.930**^†^** | **-0.268^†^** | -0.442**^†^** | **-0.056^†^** | **-0.238^†^** | -0.370**^†^** | **0.011^†^** |
| PM_2.5_ |  |  |  |  |  | 1 | **-0.214^†^** | -0.405**^†^** | **0.011^†^** | **-0.287^†^** | -0.352**^†^** | **-0.087^†^** |
| NO_X_ |  |  |  |  |  |  | 1 | 0.952**^†^** | 0.947**^†^** | 0.666**^†^** | 0.850**^†^** | **0.165^†^** |
| NO |  |  |  |  |  |  |  | 1 | 0.802**^†^** | 0.739**^†^** | 0.891**^†^** | **0.240^†^** |
| NO_2_ |  |  |  |  |  |  |  |  | 1 | 0.520**^†^** | 0.716**^†^** | **0.069^†^** |
| THC |  |  |  |  |  |  |  |  |  | 1 | 0.814**^†^** | 0.761**^†^** |
| NMHC |  |  |  |  |  |  |  |  |  |  | 1 | **0.243^†^** |
| CH_4_ |  |  |  |  |  |  |  |  |  |  |  | 1 |

CO_2_, carbon dioxide; CO, carbon monoxide; CH_4_, methane; NMHC, nonmethane hydrocarbons; NO, nitrogen monoxide; NO_2_, nitrogen dioxide; NO_X_, nitrogen oxides; O_3_, ozone; PM_10_, particulate matter < 10 μm in size; PM_2.5_, particulate matter < 2.5 μm in size; SO_2_, sulfur dioxide; THC, total hydrocarbons.

†Correlation significant at the 0.001 level (two-tailed).

Absolute values of correlation coefficient values of < 0.3 denote a low strength of correlation, which qualifies as the controlling pollutant in multiple-pollutant models of targeted pollutants.

**Supplementary Table S2.** Mean and distribution of air pollutants linked to the present study population according to the postal code over a 10-year exposure period

|  | Mean | SD | Median | 5th | 95th | Min | Max | IQR | T_1_/T_2_  cutoff | T_2_/T_3_  cutoff |
| --- | --- | --- | --- | --- | --- | --- | --- | --- | --- | --- |
| SO_2_ (ppb) | 4.16 | 1.18 | 3.85 | 2.93 | 7.03 | 0.58 | 10.62 | 0.63 | 3.59 | 4.04 |
| CO_2_ (ppm) | 401.82 | 9.40 | 399.94 | 388.45 | 418.07 | 343.47 | 463.65 | 7.60 | 398.96 | 402.95 |
| CO (ppm) | 0.54 | 0.11 | 0.53 | 0.37 | 0.69 | 0.27 | 1.17 | 0.18 | 0.47 | 0.60 |
| O_3_ (ppb) | 28.61 | 1.93 | 28.21 | 26.46 | 31.73 | 18.69 | 46.23 | 2.18 | 27.77 | 29.11 |
| PM_10_ (μg/m^3^) | 54.61 | 9.21 | 54.62 | 44.26 | 70.75 | 31.04 | 81.54 | 12.19 | 48.00 | 56.96 |
| PM_2.5_ (μg/m^3^) | 32.69 | 6.28 | 32.63 | 25.85 | 45.44 | 0.64 | 107.00 | 9.38 | 27.81 | 35.13 |
| NO_X_ (ppb) | 25.56 | 7.10 | 24.75 | 15.12 | 36.22 | 5.35 | 53.11 | 12.19 | 21.17 | 29.25 |
| NO (ppb) | 7.46 | 3.83 | 6.36 | 2.90 | 14.88 | 0.90 | 26.04 | 6.21 | 4.81 | 8.16 |
| NO_2_ (ppb) | 18.10 | 3.64 | 18.52 | 11.88 | 22.94 | 4.35 | 28.23 | 5.90 | 16.34 | 20.40 |
| THC (ppm) | 2.25 | 0.13 | 2.23 | 2.05 | 2.43 | 1.72 | 3.21 | 0.18 | 2.17 | 2.32 |
| NMHC (ppm) | 0.29 | 0.09 | 0.27 | 0.15 | 0.45 | 0.04 | 0.95 | 0.10 | 0.25 | 0.33 |
| CH_4_ (ppm) | 1.96 | 0.08 | 1.96 | 1.85 | 2.12 | 1.51 | 2.67 | 0.08 | 1.93 | 1.98 |

SD, standard deviation; 5^th^, 5 percentile; 95^th^, 95 percentile; Min, minimum; Max, maximum; IQR, interquartile range; T_1_/T_2_ cutoff, 33.33 percentile; T_2_/T_3_ cutoff, 66.66 percentile; ppb, parts per billion; ppm, parts per million; μg/m^3^, microgram/cubic meter; CO_2_, carbon dioxide; CO, carbon monoxide; CH_4,_ methane; NMHC, nonmethane hydrocarbons; NO, nitrogen monoxide; NO_2,_ nitrogen dioxide; NOx, nitrogen oxides; O_3_, ozone; PM_10_, particulate matter < 10 μm in size; PM_2.5,_ particulate matter < 2.5 μm in size; SO_2_, sulfur dioxide; THC, total hydrocarbons.

| **Supplementary Table S3.** Sensitivity analysis showing adjusted hazard ratios of developing urinary bladder cancer stratified by sex during long-term THC or NMHC exposure at a standard deviation (SD) increment controlled for PM_2.5_ and other air pollutants | | | |
| --- | --- | --- | --- |
| **Ambient pollutant category** | **Controlling pollutant***^a^* | **Adjusted HR***^b^* **(95% CI)** | |
|  |  | **Male**  **(n = 297,233)** | **Female**  **(n = 291,902)** |
| THC  (0.13-ppm increase) | - | 1.78 (1.68, 1.88) ^‡^ | 1.91 (1.78, 2.06) ^‡^ |
|  | SO_2_ | 1.76 (1.66, 1.86) ^‡^ | 1.91 (1.77, 2.05) ^‡^ |
|  | PM_10_ | 1.97 (1.86, 2.09) ^‡^ | 2.09 (1.93, 2.26) ^‡^ |
|  | PM_2.5_ | 2.04 (1.92, 2.17) ^‡^ | 2.19 (2.02, 2.37) ^‡^ |
| NMHC  (0.09-ppm increase) | - | 1.34 (1.27, 1.41) ^‡^ | 1.43 (1.33, 1.53) ^‡^ |
|  | SO_2_ | 1.31 (1.24, 1.38) ^‡^ | 1.43 (1.33, 1.54) ^‡^ |
|  | CH_4_ | 1.12 (1.07, 1.18) ^‡^ | 1.14 (1.07, 1.22) ^‡^ |
|  | SO_2_, CH_4_ | 1.08 (1.03, 1.14) ^‡^ | 1.13 (1.05, 1.21) ^‡^ |
| HR, hazard ratio; CI, confidence interval; SO_2_, sulfur dioxide; PM_10_, particulate matter < 10 μm in size; PM_2.5_, particulate matter < 2.5 μm in size; CH_4_, methane.  *^a^*Additional pollutants were added into the pollutant models for multiple analysis only when \|Pearson’s correlation coefficient\| < 0.3 (Suppl Table S1).  *^b^*Cox regression models were adjusted for age, sex, level of urbanization, black-foot disease endemic region, essential hypertension, chronic cystitis, smoking-related diagnoses, alcohol use disorders, morbid obesity, spinal cord injury, chronic liver disease, diabetes mellitus, gout, chronic kidney disease, pesticide exposures, dyslipidemia, lag0-2, season, and ambient temperature, controlled pollutants (weak correlation with THC or NMHC).  ^‡^p < 0.001. | | | |

| **Supplementary Table S4.** Sensitivity analysis showing adjusted hazard ratios of the incidence of urinary bladder cancer stratified by diabetes mellitus status during long-term THC or NMHC exposure at a standard deviation (SD) increment controlled for PM_2.5_, and other air pollutants | | | |
| --- | --- | --- | --- |
| **Air pollutant category** | **Controlling pollutant***^a^* | **Adjusted HR***^b^* **(95% CI)** | |
|  |  | **Diabetes mellitus**  **(n = 138,203)** | **Non-diabetes mellitus**  **(n = 450,932)** |
| THC  (0.13-ppm increase) | - | 1.99 (1.84, 2.16) ^‡^ | 1.76 (1.67, 1.86) ^‡^ |
|  | SO_2_ | 1.99 (1.83, 2.15) ^‡^ | 1.74 (1.65, 1.84) ^‡^ |
|  | PM_10_ | 2.20 (2.02, 2.39) ^‡^ | 1.94 (1.83, 2.05) ^‡^ |
|  | PM_2.5_ | 2.34 (2.14, 2.55) ^‡^ | 2.00 (1.88, 2.11) ^‡^ |
| NMHC  (0.09-ppm increase) | - | 1.48 (1.36, 1.60) ^‡^ | 1.33 (1.27, 1.40) ^‡^ |
|  | SO_2_ | 1.48 (1.37, 1.61) ^‡^ | 1.31 (1.24, 1.38) ^‡^ |
|  | CH_4_ | 1.22 (1.13, 1.32) ^‡^ | 1.10 (1.04, 1.15) ^‡^ |
|  | SO_2_, CH_4_ | 1.20 (1.11, 1.30) ^‡^ | 1.06 (1.01, 1.12) ^‡^ |
| HR, hazard ratio; CI, confidence interval; SO_2_, sulfur dioxide; PM_10_, particulate matter < 10 μm in size; PM_2.5_, particulate matter < 2.5 μm in size; CH_4_, methane.  *^a^*Additional pollutants were added into the pollutant models for multiple analysis only when the absolute value of Pearson’s correlation coefficient was < 0.3.  *^b^*Cox regression models were adjusted for age, sex, level of urbanization, black-foot disease endemic region, essential hypertension, chronic cystitis, smoking-related diagnoses, alcohol use disorders, morbid obesity, spinal cord injury, chronic liver disease, diabetes mellitus, gout, chronic kidney disease, pesticide exposures, dyslipidemia, lag0-2, season, and ambient temperature, controlled pollutants (weak correlation with THC or NMHC).  ^‡^p < 0.001. | | | |
